# The Association of Fatalistic Determinism With Problematic Smartphone Use in Nurses: A Cross‐Sectional Study Assessing the Mediating Roles of Self‐Control and Grit

**DOI:** 10.1155/jonm/6614190

**Published:** 2026-08-26

**Authors:** Zhuqing Feng, Wei Liu, Song Wang

**Affiliations:** ^1^ Department of Radiology, West China Hospital, Sichuan University, Chengdu, China, scu.edu.cn; ^2^ College of Teacher Education, Sichuan University of Arts and Science, Dazhou, China, sasu.cn; ^3^ School of Education, Yunnan Minzu University, Kunming, China, ynni.edu.cn; ^4^ West China School of Nursing, Sichuan University, Chengdu, China, scu.edu.cn

**Keywords:** fatalistic determinism, grit, nurses, problematic smartphone use, self-control

## Abstract

**Background:**

Problematic smartphone use (PSU) poses a significant risk to nurses’ well‐being and may have potential implications for patient safety. Although fatalistic determinism may contribute to addictive behaviors, its relation to PSU and the underlying psychological mechanisms remain unclear. This study examined the association between fatalistic determinism and PSU among nurses and the potential mediating roles of self‐control and grit.

**Methods:**

This quantitative, cross‐sectional study collected data from nurses in several hospitals in Sichuan Province, China, between July and August 2025. Participants completed validated questionnaires measuring fatalistic determinism, self‐control, grit, and PSU. Statistical analyses began with descriptive statistics, correlation testing, and a check for common method bias, followed by multiple mediation modeling to examine the direct and indirect effects (statistical associations) of the study variables.

**Results:**

A total of 429 registered nurses (mean age = 34.46 years, range = 19–60; 93.7% female) participated in this study. Fatalistic determinism showed a moderate‐to‐large correlation with PSU (*r* = 0.37, *p* < 0.001). Self‐control and grit both independently and statistically mediated this relationship (all *β* > 0.03, 95% confidence intervals [CIs]: [0.01, 0.17]). Critically, both sequential mediation pathways were found to be statistically significant: Model 1 (specifying self‐control before grit: *β* = 0.04, 95% CI: [0.01, 0.07]) and Model 2 (specifying grit before self‐control: *β* = 0.07, 95% CI: [0.03, 0.11]). All findings persisted even after controlling for sociodemographic factors and job stress.

**Conclusion:**

Fatalistic determinism was associated with PSU among nurses, and this association showed indirect effects through both self‐control and grit. The interrelated nature of these variables suggests that interventions targeting either self‐regulatory domain may have synergistic benefits.

**Implications for Nursing Management:**

To mitigate PSU in nursing staff, nurse managers and leaders should consider implementing multilevel strategies that address maladaptive fatalistic beliefs while simultaneously fostering impulse control and long‐term goal commitment.

## 1. Introduction

The rapid proliferation of smartphones has fundamentally altered daily life, offering unprecedented connectivity and access to information. However, this ubiquity has brought with it a growing public health concern: problematic smartphone use (PSU) [1], which is characterized by excessive and compulsive smartphone use leading to functional impairment. PSU is increasingly recognized as a behavioral addiction with detrimental effects on mental health, sleep quality, and work performance [2, 3]. Within the high‐stress environment of healthcare, this issue takes on critical importance. For nurses, whose professional demands require sustained focus, emotional labor, and quick decision‐making, PSU may have the potential to compromise patient safety, reduce work efficiency, and exacerbate stress, anxiety, and burnout [4, 5]. Understanding the psychosocial antecedents of PSU in this population is, therefore, a pressing priority for nursing management and practice.

One promising, yet underexplored, individual psychosocial antecedent is fatalistic determinism. This worldview involves the belief that life events are predetermined by fate, chance, or external forces beyond personal control [6, 7]. People with strong fatalistic beliefs tend to perceive their actions as inconsequential in shaping their future. This cognitive orientation has been linked to negative mental health outcomes, including depression, anxiety, stress, passive coping styles, and risky behaviors [8–11]. In the context of behavioral addictions, fatalism may create a vulnerability; if an individual feels powerless over their life circumstances, they may be less likely to resist immediate gratifications, such as smartphone use, thereby increasing the risk of addiction [12]. This aligns with the broader literature identifying maladaptive coping mechanisms as central to PSU [13]. However, the specific mechanism through which fatalistic determinism translates into addictive behaviors among nurses remains largely unexamined.

A potential explanatory pathway involves self‐regulatory resources, specifically self‐control and grit. These are conceptually related yet distinct constructs, both fundamentally involving the alignment of actions with intended goals [14]. Self‐control, the capacity to override impulses and regulate thoughts, emotions, and behaviors in the presence of temptation [15], is a well‐established protective factor against addictive behaviors [16]. Individuals with high self‐control are better equipped to resist the urge to check their smartphones inappropriately [17]. Grit—the combination of perseverance and passion for long‐term objectives—is defined by the persistent drive toward major goals despite challenges [18] and is associated with higher resilience and hardiness [19]. This robust character suggests that gritty individuals employ more adaptive strategies for navigating negative experiences, thereby reducing their tendency to use smartphones problematically for emotional compensation [20]. Moreover, the superior sustained attention found in gritty individuals shields them from digital distractions, limiting problematic smartphone engagement [21]. Crucially, research increasingly positions grit as a stable factor that protects against the emergence of PSU [22, 23].

Drawing upon the interaction of person‐affect‐cognition‐execution (I‐PACE) model [24], we propose that fatalistic determinism acts as a distal predisposing factor that compromises self‐regulatory resources, which in turn increases susceptibility to PSU. The I‐PACE model posits that specific addictive disorders or behaviors result from interactions between predisposing vulnerabilities (e.g., fatalistic determinism) and mediating affective‐cognitive components (e.g., self‐control and grit). Within this framework, distal factors like fatalistic determinism beliefs may compromise an individual’s executive functions and cognitive control, which then facilitates addictive behaviors as a maladaptive coping strategy [25]. Specifically, fatalistic determinism—characterized by a belief in external control—is inherently at odds with the internal agency required for effective self‐regulation and sustained goal pursuit [26]. This aligns with prior research showing that fatalistic determinism is negatively associated with self‐control and grit [8, 27].

Moreover, it is plausible that self‐control and grit act as sequential mediators in the relationship between fatalistic determinism and PSU. However, the sequential interplay between these two self‐regulatory resources remains theoretically ambiguous, presenting two competing possibilities. On one hand, fatalistic determinism may first diminish an individual’s general capacity for self‐control—the ability to resist immediate impulses—which in turn undermines the specific, long‐term perseverance required by grit, ultimately linking to addictive phone use. This pathway is consistent with evidence that impaired self‐control is associated with addictive behaviors [16], while low grit may reflect a broader disengagement from meaningful goals that exacerbates vulnerability [28]. On the other hand, a lack of grit—the inability to stay committed to meaningful goals—might deplete one’s motivation to exercise daily self‐control, creating a separate pathway to addiction. In other words, without passion and perseverance for long‐term objectives, individuals may lack the motivational basis to exert moment‐to‐moment self‐control against digital temptations [14]. To date, no study has examined these competing sequential mediation models to establish the joint link between fatalistic determinism, self‐control, grit, and PSU.

### 1.1. The Present Study

While previous research has investigated associations among fatalistic determinism, self‐control, grit, and nurse outcomes separately, the specific mechanism through which fatalistic determinism is linked to addictive behaviors among nurses remains largely unexamined. To our knowledge, no study has examined the potential mediating roles of self‐control and grit in the relationship between fatalistic determinism and PSU among the nursing population. Therefore, this study aims to investigate the association between fatalistic determinism and PSU in nurses, with a specific focus on unpacking the mediating roles of self‐control and grit. Critically, we investigate two competing sequential mediation models to explore the potential ordering of these self‐regulatory resources based on theoretical reasoning, while acknowledging that cross‐sectional data cannot establish definitive temporal precedence. Based on the existing literature and theoretical reasoning, we propose the following hypotheses: H1: Fatalistic determinism is positively linked to nurses’ PSU. H2: Self‐control mediates the link between fatalistic determinism and PSU. H3: Grit mediates the link between fatalistic determinism and PSU. H4a: Self‐control and grit sequentially mediate the link between fatalistic determinism and PSU, with self‐control specified as the first mediator followed by grit (theoretically plausible pathway 1). H4b: Grit and self‐control sequentially mediate the link between fatalistic determinism and PSU, with grit specified as the first mediator followed by self‐control (theoretically plausible pathway 2). We test both competing models to explore potential pathways, acknowledging that cross‐sectional data cannot establish definitive temporal precedence.

By examining these competing mediation models, we hope to provide insights into the potential psychological mechanisms that link a fatalistic worldview to PSU among nurses. The findings may have the potential to inform the development of targeted interventions aimed at modifying maladaptive beliefs and strengthening self‐regulatory resources, ultimately reducing PSU and promoting healthier digital habits in the nursing workforce. Given the growing global recognition of digital dependency as an occupational health threat among healthcare workers [29], elucidating these mechanisms may be essential for developing effective strategies to safeguard both nurse well‐being and patient care quality.

## 2. Methods

### 2.1. Study Design

We adopted a quantitative approach using a cross‐sectional survey to investigate the associations among key research variables. The main goal was to test two theoretically derived sequential mediation models, determining the extent to which self‐control and grit (acting alone or consecutively) mediate the link between fatalistic determinism and PSU. The procedures and presentation of findings were aligned with the STROBE checklist for cross‐sectional studies [30].

### 2.2. Sampling and Procedure

This study used convenience sampling to recruit registered nurses from two secondary and two tertiary public hospitals in Sichuan Province, China. The hospitals were purposively selected based on existing research collaborations and their willingness to participate, with the aim of ensuring institutional diversity. To safeguard participant anonymity, we did not collect information regarding departmental affiliation. While we recognized that workplace factors—such as team dynamics, patient acuity, and clinical flow—may impact the study variables, capturing this level of detail posed a risk of reidentification, particularly in smaller specialized units like the intensive care or emergency departments. Data were gathered online through the wjx.cn survey tool over July and August 2025, facilitated by on‐site, trained research staff. Eligibility required participants to possess a current Chinese registered nurse license, at least 12 months of continuous professional clinical experience, and agreement to the informed consent terms. Students and those in provisional nursing training were ineligible. Out of 448 nurses initially approached, 429 provided complete and usable data, achieving a response rate of 95.75%. The 19 discarded questionnaires were removed due to significant incompleteness, obvious response patterns (such as identical answers across items), or evidence of nonattentive completion. To assess potential nonresponse bias, we compared the demographic characteristics of the 19 excluded participants with those of the final sample using available demographic information. No significant differences were found in sex (*χ* ^2^ = 0.54, *p* = 0.463), age (*t* = 0.52, *p* = 0.602), income (*t* = −0.18, *p* = 0.859), years of education (*t* = −1.65, *p* = 0.099), or length of nursing work (*t* = 0.97, *p* = 0.332), suggesting that nonresponse did not substantially bias the sample.

The required sample size was determined a priori using power analysis for mediation models with bootstrap procedures [31]. Based on the assumption of a small‐to‐medium effect size (*f* ^2^ = 0.05) for the proposed mediation model, with *α* = 0.05, power = 0.80, and 3 predictor variables in the full model, the required sample size was calculated as 303 participants using G^∗^ Power software (Version: 3.1) [32]. To account for potential incomplete responses (estimated at 15%–20%) and to ensure adequate power for the bootstrap analyses, we aimed to recruit approximately 400 participants. The final sample of 429 participants exceeded the minimum requirement, providing sufficient statistical power to detect the hypothesized effects.

### 2.3. Variables and Measures

#### 2.3.1. Fatalistic Determinism

Participants’ fatalistic beliefs were assessed with the five‐item fatalistic determinism subscale of the Free Will and Determinism Plus Scale [6]. Responses were captured using a 7‐point Likert scale, ranging from 1 (*strongly disagree*) to 7 (*strongly agree*), with higher aggregated scores reflecting a greater tendency of fatalistic determinism. The Chinese version of this scale was translated and backtranslated by the authors of the current study [27], and its reliability and validity have been demonstrated in different Chinese populations [8, 33]. In the present dataset, the scale exhibited satisfactory internal consistency (Cronbach’s *α* = 0.87).

#### 2.3.2. Self‐Control

Self‐control capacity was evaluated using the Brief Self‐Control Scale (BSCS), developed by Tangney et al. (2004) [15]. This instrument assesses self‐control as a single‐dimensional construct, consisting of 13 distinct items. Participants expressed their agreement with the statements on a 5‐point response continuum, ranging from 1 (“*strongly disagree*”) to 5 (“*strongly agree*”). A total score was calculated by adding up all the responses to the items, with higher scores indicating a stronger level of self‐control. The BSCS is known for its established reliability and validity, successfully measuring self‐control, including within Chinese contexts and specifically among nursing cohorts [34, 35]. In the present dataset, the BSCS exhibited adequate internal consistency (Cronbach’s *α* = 0.82).

#### 2.3.3. Grit

Individuals’ grit levels were determined using the 8‐item Short Grit Scale (SGS), employing the Chinese adaptation validated by Li et al. [36], which stems from the original work by Duckworth and Quinn [37]. The SGS measures two distinct facets: sustained interest and persistent effort. Participants rated how well each item described them on a 5‐point Likert scale (1 = “*not at all like me*” to 5 = “*very much like me*”). The overall grit score was derived by summing the scores across all eight items, meaning higher totals indicated elevated levels of grit. This scale has demonstrated established reliability and validity within Chinese samples, including research focused on nursing samples [34, 38]. In the present dataset, the SGS exhibited adequate internal consistency (Cronbach’s *α* = 0.72).

#### 2.3.4. PSU

Participants’ PSU levels were quantified using the 10‐item Short Version Smartphone Addiction Scale (SAS‐SV) [39]. This tool functions as a single‐factor screening measure assessing PSU behaviors. Agreement levels were indicated by respondents using a 6‐point Likert scale, anchored from 1 (“*strongly disagree*”) to 6 (“*strongly agree*”). The total score is the sum of all item responses, where greater totals denote a higher severity of self‐reported PSU. It is important to note that this instrument is a screening scale and does not constitute a clinical diagnosis of addiction. The Chinese SAS‐SV has been translated and validated by Zhao et al. [40], and it exhibits sound psychometric properties and is widely adopted in studies involving Chinese populations, notably within nursing research [41, 42]. In the present dataset, the SAS‐SV exhibited satisfactory internal consistency (Cronbach’s *α* = 0.92).

#### 2.3.5. Job Stress

To examine the potential confounding effect of occupational stress on the links between fatalistic determinism, self‐control, grit, and PSU, the Job Stress Scale (JSS) [43] was administered. This instrument is unidimensional and consists of four items, each answered on a five‐point Likert scale from “*strongly disagree*” (1) to “*strongly agree*” (5). A total job stress score was calculated by summing the responses to all four items, where higher scores reflect increased levels of perceived job stress. Prior validation studies have confirmed the JSS’s good psychometric properties in the Chinese population [44]. In the present dataset, the JSS demonstrated satisfactory internal consistency, yielding a Cronbach’s alpha of 0.72.

#### 2.3.6. Demographic and Professional Variables

Participants provided information on key characteristics, including age, sex, annual income, years of nursing employment, formal educational attainment, marital status, and professional rank. This set of variables served dual functions: they were utilized to describe the sample demographics, and they were subsequently entered into the adjusted analyses as covariates.

### 2.4. Statistical Analysis

Statistical analysis was conducted utilizing IBM SPSS Statistics 26.0 alongside the PROCESS macro 4.1 [45]. First, descriptive statistics—specifically the mean/median, standard deviation/interquartile range, range, frequency, skewness, and kurtosis—were generated for all demographic and research variables. The next step involved assessing the potential for common method bias (CMB) in the self‐report data via Harman’s single‐factor test [46]. This required performing an exploratory factor analysis across all items. Consistent with established criteria, CMB was considered nonsignificant if one factor explained under 40% of the total extracted variance [46]. Finally, preliminary associations between the study variables were explored using bivariate Pearson correlation coefficients.

Subsequently, two competing multiple mediation models were tested to elucidate the mediating roles of self‐control and grit between fatalistic determinism (independent variable, *X*) and PSU (dependent variable, *Y*). The sequence of mediator variables (*M*) was varied: Model 1 specified self‐control as the first mediator followed by grit, while Model 2 reversed this order. The PROCESS Model 6 [45] provided the structure for analyzing both the direct effect (*X* ⟶ *Y*) and the indirect effects involving self‐control (M1/M2) and grit (M2/M1) operating in parallel and serially. Statistical inference for the indirect effects relied on bias‐corrected bootstrapping, executing 5000 resamples. Significance was established if the resulting 95% confidence interval (CI) excluded zero. To evaluate the stability of these mediation findings, we performed supplementary analyses in which sociodemographic factors (e.g., age, sex, accumulated years of education, income level, and duration of nursing employment) and job stress were treated as statistical covariates.

### 2.5. Ethical Standards

Ethical approval for this study was obtained from the Ethics Committee of West China Hospital, Sichuan University (Approval No.: 2021‐1216; Approval Date: November 4, 2021). This approval covered the study protocol and data collection period (July–August 2025). Before survey completion, all prospective respondents were briefed via an information sheet covering the research aims, methodology, expected benefits, and confidentiality commitments. Attendance was voluntary, and written informed consent was obtained from every participant. To guarantee anonymity, all collected data were stripped of personal identifiers, meaning no identifying information was recorded with the final responses.

## 3. Results

### 3.1. Participant Characteristics

As shown in Table 1, a total of 429 nurses participated in this study. The mean age of participants was 34.46 years, ranging from 19 to 60 years. The majority of participants were female (93.7%). Regarding educational level, most nurses held a bachelor’s degree (78.3%), followed by college degree (19.6%). More than half of the participants were married (67.8%), and the most common professional title was intermediate (47.1%). The mean years of education was 15.80 (range: 12–19), and the average annual income was 84,500 CNY (range: 20,000–260000). Participants had an average of 12.74 years of nursing experience (range: 1–41).

**TABLE 1:** Sociodemographic characteristics of the participants.

Variable	Mean/median ± SD/IQR (range) *N*%
Age (years)	34.46 ± 8.14 (19–60)
Sex	
Female	402 (93.7%)
Male	27 (6.3%)
Educational level	
Graduate degree	5 (1.2%)
Bachelor degree	336 (78.3%)
College degree	84 (19.6%)
Secondary vocational degree	4 (0.9%)
Marital status	
Married	291 (67.8%)
Unmarried	118 (27.5%)
Divorced	15 (3.5%)
Widowed	3 (0.7%)
Other	2 (0.5%)
Professional title	
Senior	4 (0.9%)
Vice senior	32 (7.5%)
Intermediate	202 (47.1%)
Primary	183 (42.6%)
None	8 (1.9%)
Years of education	16 ± 0 (12–19)
Income (CNY/year)	80,000 ± 40,000 (20,000–2,60,000)
Length of nursing work (years)	12.74 ± 8.62 (1–41)

*Note:* N, number. The mean and standard deviation (SD) were used for normally distributed continuous variables (age and length of nursing work); the median and interquartile range (IQR) were used for nonnormally distributed continuous variables (years of education and income).

### 3.2. CMB Assessment

The exploratory factor analysis revealed that the interrelationships among the study variables were distributed across multiple dimensions, rather than being dominated by a single underlying construct. A total of six factors were extracted, each possessing an eigenvalue exceeding unity. Importantly, the variance explained by the most salient factor was limited, accounting for only 28.32% of the total variance. Since this figure falls short of the 40% conventional threshold used to confirm strong common factor influence, the data may be unlikely to suffer from substantial CMB.

### 3.3. Descriptive Statistics and Bivariate Correlations

Table 2 presents the descriptive statistics and bivariate correlations among the study variables. All variables demonstrated acceptable levels of skewness and kurtosis (values between −1 and +1), indicating normal univariate distributions [47]. Bivariate correlation analysis revealed statistically significant relations between all constructs. Specifically, fatalistic determinism was positively linked with PSU (*r* = 0.37, *p* < 0.001). Conversely, it was negatively correlated with self‐control (*r* = −0.37, *p* < 0.001) and grit (*r* = −0.38, *p* < 0.001). Self‐control and grit were positively correlated (*r* = 0.68, *p* < 0.001). PSU was negatively associated with both self‐control (*r* = −0.48, *p* < 0.001) and grit (*r* = −0.45, *p* < 0.001).

**TABLE 2:** Descriptive statistics and bivariate correlations of study variables.

Measure	Mean ± SD	Skewness	Kurtosis	1	2	3	4
1. Fatalistic determinism	19.74 ± 7.01	−0.04	−0.36	—			
2. Self‐control	44.72 ± 8.11	0.15	−0.54	−0.37	—		
3. Grit	27.63 ± 4.44	0.97	0.48	−0.38	0.68	—	
4. Problematic smartphone use	29.36 ± 11.99	0.36	−0.06	0.37	−0.48	−0.45	—
5. Job stress	11.64 ± 3.49	−0.20	−0.49	0.18	−0.27	−0.30	0.17

*Note:* All correlation coefficients were statistically significant at the *p* < 0.001 level. SD, standard deviation.

### 3.4. Mediation Associations Between Study Variables

**FIGURE 1:**
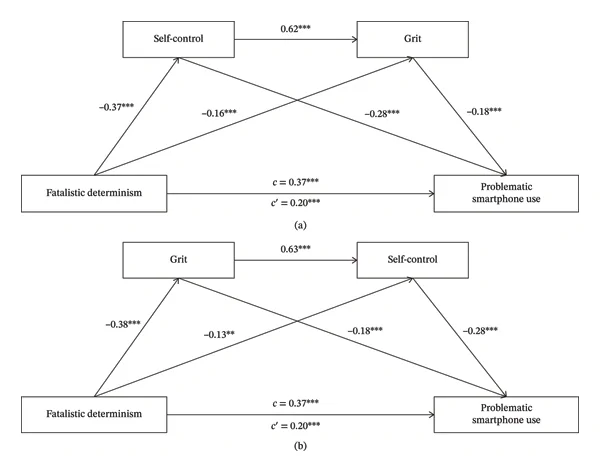
Fatalistic determinism is linked to problematic smartphone use through the self‐control ⟶ grit pathway (a) or the grit ⟶ self‐control pathway (b). Standardized regression coefficients were displayed in the path diagram; *c*, total effect; *c*′, direct effect. ^∗∗∗^, *p* < 0.001; ^∗∗^, *p* < 0.01; ^∗^, *p* < 0.05.

To examine the mediating roles of self‐control and grit in the relationship between fatalistic determinism and PSU, we tested two competing mediation models with different sequential orders of the mediators (Model 1 [self‐control as the first mediator followed by grit] vs. Model 2 [grit as the first mediator followed by self‐control]). The results of the mediation analysis are presented in Table 3 and illustrated in Figure 1. For both proposed models, the total effect of fatalistic determinism on PSU was statistically significant (*β* = 0.37, 95% CI: [0.29, 0.46]). After controlling for the proposed mediators, the direct effect of fatalistic determinism on PSU remained statistically significant (*β* = 0.20, 95% CI: [0.12, 0.29]). This indicates partial mediation in the models, and the total indirect effect was 0.17 (95% CI: [0.12, 0.23]).

**TABLE 3:** Total, direct, and indirect effects of the mediation models.

Effect	Effect size (*β*)	Bootstrap SE	Bootstrap 95% CI
Model 1 (specifying self‐control before grit)			
Total effect (Fatalistic determinism ⟶ Problematic smartphone use)	0.37	0.04	[0.29, 0.46]
Direct effect (Fatalistic determinism ⟶ Problematic smartphone use)	0.20	0.04	[0.12, 0.29]
Indirect effect	0.17	0.03	[0.12, 0.23]
Fatalistic determinism ⟶ Self‐control ⟶ Problematic smartphone use	0.10	0.03	[0.05, 0.17]
Fatalistic determinism ⟶ Grit ⟶ Problematic smartphone use	0.03	0.01	[0.01, 0.05]
Fatalistic determinism ⟶ Self‐control ⟶ Grit ⟶ Problematic smartphone use	0.04	0.01	[0.01, 0.07]
Model 2 (specifying grit before self‐control)			
Total effect (Fatalistic determinism ⟶ Problematic smartphone use)	0.37	0.04	[0.29, 0.46]
Direct effect (Fatalistic determinism ⟶ Problematic smartphone use)	0.20	0.04	[0.12, 0.29]
Indirect effect	0.17	0.03	[0.12, 0.23]
Fatalistic determinism ⟶ Grit ⟶ Problematic smartphone use	0.07	0.02	[0.02, 0.12]
Fatalistic determinism ⟶ Self‐control ⟶ Problematic smartphone use	0.03	0.01	[0.01, 0.07]
Fatalistic determinism ⟶ Grit ⟶ Self‐control ⟶ Problematic smartphone use	0.07	0.02	[0.03, 0.11]

Abbreviations: CI, confidence interval; SE, standard error.

For Model 1, the indirect effect was decomposed into three pathways. First, self‐control significantly mediated the link between fatalistic determinism and PSU (*β* = 0.10, 95% CI: [0.05, 0.17]). Grit also served as a significant mediator (*β* = 0.03, 95% CI: [0.01, 0.05]). Notably, the serial mediation pathway from fatalistic determinism to self‐control to grit to PSU was significant (*β* = 0.04, 95% CI: [0.01, 0.07]). For Model 2, the total indirect effect was equal to that for Model 1, but the specific indirect effects differed in magnitude. Grit significantly mediated the link between fatalistic determinism and PSU (*β* = 0.07, 95% CI: [0.02, 0.12]), and self‐control also served as a significant mediator (*β* = 0.03, 95% CI: [0.01, 0.07]). The serial mediation pathway from fatalistic determinism to grit to self‐control to PSU was significant (*β* = 0.07, 95% CI: [0.03, 0.11]).

Comparing the sequential mediation effects, Model 2 showed a slightly larger indirect effect than Model 1, when considering the isolated three‐step mediation components shown in Table 3. However, given the cross‐sectional nature of the data, this difference should not be interpreted as evidence of one pathway’s superiority. Critically, both sequential orderings were significant, suggesting that self‐control and grit may be dynamically interrelated in their response to fatalistic beliefs, with potential bidirectional associations that cannot be disentangled with the current design. After controlling for sociodemographic factors (sex, age, years of education, income, and length of nursing work), these results were generally consistent with the main analyses (for details, see Table S1 and Figure S1). Even when job stress was treated as a covariate in the mediation models, the results remained robust, with effect sizes comparable to those in the main analyses (for details, see Table S2 and Figure S2). Finally, to control for potential CMB, we reran our mediation modeling analyses by using the first factor from the exploratory factor analysis as a covariate [46]. These sensitivity analyses yielded results that were consistent with our main findings, despite some reduction in the magnitude of the effects (for details, see Table S3 and Figure S3).

## 4. Discussion

This study sought to investigate the link between fatalistic determinism and PSU among nurses, with a specific focus on the mediating roles of self‐control and grit, both individually and sequentially. The results largely support our hypotheses, revealing a significant positive relation between fatalistic determinism and PSU. Importantly, this relationship was partially mediated by self‐control and grit, both separately and in two competing sequential manners. These findings offer valuable insights into the psychological mechanisms by which a fatalistic worldview is linked to PSU and have implications for nursing management, mental health support, and the design of targeted interventions for nurses. It should be noted that PSU was assessed using a self‐report screening scale rather than clinical diagnostic criteria. While the SAS‐SV can measure problematic use patterns and symptom severity, it cannot substitute for a comprehensive clinical evaluation.

### 4.1. Key Findings and Interpretations

First, the observed positive relation between fatalistic determinism and PSU (H1) confirms and extends previous research linking fatalism to negative mental health outcomes and maladaptive coping behaviors [8–11]. In the high‐stress context of nursing, where work demands are substantial and control over patient outcomes can feel limited [48], a belief that life events are predetermined by external forces may lead individuals to adopt a passive coping orientation [49]. This cognitive passivity can make the immediate gratification, distraction, and emotional escape offered by smartphones particularly appealing, thereby increasing the risk of developing addictive usage patterns [12, 13]. This finding underscores the notion that cognitive orientations, such as fatalism, may be associated with behavioral addictions in the workplace, although causal inference requires longitudinal validation. The result aligns with the broader propositions of the I‐PACE model, which posits that predisposing characteristics interact with affective and cognitive responses to facilitate addictive disorders and behaviors [24]. By identifying fatalistic determinism as a relevant predisposing factor within the nursing population, this study extends the applicability of the I‐PACE model to occupational health contexts and highlights the need to consider worldview‐level variables in understanding PSU in healthcare professionals.

The finding that self‐control mediates the link between fatalistic determinism and PSU (H2) is consistent with a large body of literature identifying self‐control as a key protective factor against addictive behaviors [16, 17]. Fatalistic determinism, characterized by a belief in external control, is fundamentally at odds with the internal agency required for effective self‐control [50]. Nurses who perceive their lives as controlled by fate or external circumstances may possess diminished motivation to exert the effortful cognitive control needed to resist the impulse to check their smartphones, particularly during moments of workplace boredom, emotional exhaustion, or between demanding patient interactions. This association with lower self‐control may contribute to compulsive use, as the individual is left without the primary psychological mechanism needed to curb automatic behavioral responses [51]. This mediation pathway provides empirical support for the theoretical assertion within the I‐PACE model that distal predisposing factors exert their influence on addictive behaviors by compromising domain‐general inhibitory control mechanisms [25]. Overall, the finding suggests that fatalistic beliefs do not directly translate into addictive behaviors but rather operate by degrading the self‐control capacity that would, otherwise, serve as a protective barrier.

Similarly, the mediation effect of grit on the relation between fatalistic determinism and PSU (H3) highlights the role of long‐term goal commitment as a protective factor. Grit, defined as passion and perseverance for long‐term goals, enables individuals to maintain focus on meaningful objectives and resist distracting impulses [18, 21]. A fatalistic worldview, however, can fundamentally undermine this capacity by fostering a sense that sustained effort toward long‐term goals is ultimately futile, as outcomes are already predetermined. This cognitive orientation can diminish a nurse’s drive to persist in their demanding career, pursue professional development, or maintain commitment to personal growth, making them more susceptible to the immediate, low‐effort rewards of smartphone use as a form of disengagement from an apparently meaningless trajectory [52]. Without the guiding direction provided by long‐term goals, the allure of digital temptations becomes more difficult to resist. This finding extends the nomological network of grit by identifying fatalistic determinism as a significant cognitive antecedent that may be associated with this valuable psychological resource [28]. It also aligns with recent work positioning grit as a protective factor against PSU [22, 23], while simultaneously revealing the belief systems that may undermine it.

The most theoretically intriguing finding of this study is the confirmation of both competing sequential mediation models (H4a and H4b). The significance of both modeling pathways suggests a dynamic and interrelated association between self‐control and grit in the context of fatalism, rather than evidence for one pathway’s superiority. This duality suggests a theoretical possibility that fatalistic beliefs may be associated with lower levels in both the motivational system (grit) and the inhibitory system (self‐control) [53], though cross‐sectional data cannot establish causal ordering. The slightly larger indirect effect observed in Model 2 (grit as the first mediator followed by self‐control) offers a potentially nuanced insight, though this difference should be interpreted cautiously given the cross‐sectional design. This pathway suggests that fatalistic beliefs may be associated with grit—when a nurse loses sight of why their work or personal growth matters, they lose a key source of motivation that fuels sustained effort—and this motivational void then makes it harder to exert the momentary, effortful self‐control needed to resist digital temptations [54]. Conversely, the significance of Model 1 implies that a chronic lack of impulse control may be associated with the capacity for long‐term commitment, as every distracting impulse is acted upon, preventing the accumulation of effort required for meaningful goal pursuit [55]. Rather than establishing one model as superior, our findings suggest that both pathways represent plausible mechanisms that may operate simultaneously in real‐world settings. This bidirectional vulnerability aligns with the compensatory internet use theory [20], which posits that individuals turn to digital engagement to escape negative experiences or unmet needs. This complexity advances theoretical understanding by demonstrating that self‐control and grit are not merely parallel mediators but are dynamically interrelated in their response to cognitive vulnerability factors.

### 4.2. Limitations and Future Research Directions

While this study provides valuable insights, several limitations must be acknowledged. First, the cross‐sectional design precludes definitive causal inferences. Although our competing models are theoretically grounded in the I‐PACE framework [24], the measurement of all variables at a single time point prevents the empirical validation of the proposed causal pathways. Consequently, the significant findings should be interpreted as plausible statistical associations rather than established sequences. Future longitudinal, experimental, or experience sampling research is necessary to confirm the temporal dynamics between fatalistic determinism, self‐control, grit, and PSU. Moreover, because variables were measured concurrently, we could not empirically distinguish between the two competing mediation models. Both models yielded significant results, yet the cross‐sectional data provide no basis to determine which sequence is superior; this limitation necessitates further investigation via longitudinal designs.

Second, the data were collected using self‐report scales, which may be subject to social desirability and CMB [46, 56]. While Harman’s single‐factor test and supplementary analyses controlling for the first factor extracted from exploratory factor analysis suggested that CMB may not be a major concern in this dataset, these statistical remedies cannot completely eliminate the possibility of shared method variance. Future research could incorporate objective measures of smartphone use (e.g., device usage tracking applications and screen time data), collateral reports from colleagues or supervisors, or multimodal assessments that combine self‐report with behavioral or physiological measures.

Third, the sample, while adequate for analysis, was recruited via convenience sampling from one province in China and was predominantly female. This limits the generalizability of the findings to more gender‐diverse nursing populations or different cultural contexts where beliefs about fate and self‐agency may differ [57]. The lack of random sampling and the geographic restriction to Sichuan Province may limit the representativeness of the findings to other regions or healthcare systems. Additionally, we did not systematically collect data on the hospital size, department distributions, nurse‐to‐patient ratios, or other institutional characteristics that may moderate the observed relations. Future research with more diverse, randomly sampled, and multiregion samples is needed to establish the generalizability of our findings.

Fourth, while the SAS‐SV is a validated screening tool for PSU, it does not provide a clinical diagnosis of addiction [39–42]. The measure captures self‐reported symptom severity and problematic use patterns but cannot substitute for comprehensive clinical evaluation. Future studies may benefit from incorporating structured clinical interviews or multimethod assessments that include both self‐report and objective behavioral measures (e.g., device usage tracking applications and screen time data). Additionally, it is important to emphasize that this study did not directly measure patient safety events or clinical outcomes. While PSU among nurses may have implications for attention and clinical decision‐making, the association between self‐reported problematic use and actual patient safety outcomes requires direct empirical investigation in future research.

Finally, the adjusted models included only basic sociodemographics and available occupational factors (e.g., job stress). Critical occupational and psychological confounders—such as detailed workload measures, shift work patterns, department types, sleep quality, anxiety, depression, and burnout—were not measured and therefore could not be controlled. These variables may be important confounders or mediators in the relationship between fatalistic determinism and PSU [3, 5]. For instance, nurses experiencing high burnout or psychological distress may be more vulnerable to both fatalistic beliefs and PSU [4, 8]. Future research should incorporate comprehensive assessments of these occupational and psychological factors to better isolate the unique contribution of fatalistic determinism and to examine potential moderated mediation effects.

## 5. Implications for Nursing Management and Practice

The findings of this study have several implications for nursing management and practice, which can be organized at individual, unit, and organizational levels. At the organizational level, healthcare institutions should consider developing comprehensive digital professionalism policies that provide clear, nonpunitive guidelines for appropriate smartphone use during work hours. These policies should balance patient safety concerns with the legitimate need for connectivity and should be developed collaboratively with nursing staff to ensure feasibility and acceptance. Unit‐level expectations should be tailored to specific clinical contexts (e.g., intensive care units may require stricter limitations than administrative settings) and communicated clearly to all staff.

Nurse managers play a critical role in implementing nonstigmatizing staff well‐being supports. Rather than singling out individuals for smartphone use, managers should frame digital wellness as a shared organizational priority, normalizing help‐seeking and reducing shame associated with problematic use. This includes establishing clear referral pathways to connect staff with mental health or addiction support services when needed, ensuring confidentiality and protecting against professional repercussions. Systematic monitoring of workload and occupational stress is essential, as these factors may contribute to maladaptive coping through smartphone use [43]. Nurse managers should regularly assess unit‐level stressors, nurse‐to‐patient ratios, and staff burnout, implementing workload adjustments when feasible to reduce the conditions that may precipitate problematic digital habits.

At the individual level, healthcare organizations should recognize that cognitive beliefs, such as fatalistic determinism, may be a potential vulnerability factor for PSU and its potential downstream effects on patient care and nurse well‐being. Workshops and psychoeducation programs could help nurses identify and challenge maladaptive fatalistic thoughts [58], fostering a greater sense of agency and internal control. Second, interventions aimed at strengthening self‐regulatory resources are crucial. Mindfulness‐based stress reduction, cognitive‐behavioral therapy, and resilience training can enhance self‐control by improving impulse regulation [59]. Simultaneously, mentorship programs, goal‐setting workshops, and career development discussions can help cultivate grit by reinforcing nurses’ passion for their profession and their perseverance through challenges [60].

Manager‐facilitated career‐development and mentorship programs represent a particularly promising strategy, as they address both the individual and organizational levels [61]. By helping nurses to articulate their long‐term professional goals and providing structured support to help them achieve these goals, managers can foster self‐control and resilience, while also demonstrating the organization’s commitment to staff well‐being and professional development.

Finally, given the interrelated nature of these factors as suggested by our cross‐sectional findings, interventions should take a holistic approach. Fostering a sense of purpose and long‐term meaning in nursing work may provide the necessary motivation for nurses to exercise the daily self‐control needed to manage their digital habits effectively. Critically, these organizational and managerial strategies should be implemented without stigma, recognizing that smartphone use is a normative behavior and that support, rather than punishment, is the most effective approach to promoting healthy digital habits among nursing staff.

## 6. Conclusion

In conclusion, this study offers novel evidence that fatalistic determinism is positively associated with PSU in nurses and that this relationship is partially and statistically explained by self‐control and grit. Critically, our findings support two competing sequential pathways, suggesting that fatalism may be linked to PSU through associations with lower levels of both self‐control and grit, which appear to be interrelated in this context. These results highlight the importance of addressing maladaptive cognitive beliefs and strengthening multiple facets of self‐regulatory resources to mitigate PSU among nursing staff, with potential downstream implications for patient safety and care quality that should be examined in future research. By understanding the complex interplay between worldview, self‐control, and grit, healthcare organizations can develop more nuanced approaches to supporting nurse well‐being. Future research should employ longitudinal designs to confirm causality and explore the efficacy of intervention programs tailored to the unique cognitive and emotional landscape of the nursing profession.

## Author Contributions

Zhuqing Feng: data curation, formal analysis, investigation, methodology, and writing–original draft. Wei Liu: investigation, methodology, validation, and writing–review and editing. Song Wang: conceptualization, project administration, resources, supervision, and writing–review and editing.

## Funding

This study was supported by the Key Research and Development Program of Sichuan Province (Grant no. 2023YFS0084).

## Disclosure

This article reports original research that has not been previously published and is not under consideration elsewhere. The data and findings in this study are new and have not been used in any prior publication. All authors have read and approved the final version of the manuscript and agreed to be accountable for all aspects of the work.

## Ethics Statement

Ethical approval for this study was obtained from the Ethics Committee of West China Hospital, Sichuan University (Approval No.: 2021‐1216; Approval Date: November 4, 2021), which covered the study protocol and data collection period (July–August 2025). All participants signed a written informed consent form, acknowledging the procedures and confirming their voluntary participation.

## Consent

No patients or members of the public were involved in the design, conduct, reporting, or dissemination plans of this research. The study focused on registered nurses as participants, and their involvement was limited to completing the survey instruments.

## Conflicts of Interest

The authors declare no conflicts of interest.

## Supporting Information

Additional supporting information can be found online in the Supporting Information section.

## Supporting information


**Supporting Information 1** Table S1. Total, direct, and indirect effects of the mediation models after controlling for sociodemographic factors. Table S2. Total, direct, and indirect effects of the mediation models after controlling for job stress. Table S3. Total, direct, and indirect effects of the mediation models after controlling for the first factor in the Harman’s single‐factor test. Figure S1. Fatalistic determinism is linked to problematic smartphone use through the self‐control ⟶ grit pathway (a) or the grit ⟶ self‐control pathway (b) after controlling for sociodemographic factors. Figure S2. Fatalistic determinism is linked to problematic smartphone use through the self‐control ⟶ grit pathway (a) or the grit ⟶ self‐control pathway (b) after controlling for job stress. Figure S3. Fatalistic determinism is linked to problematic smartphone use through the self‐control ⟶ grit pathway (a) or the grit ⟶ self‐control pathway (b) after controlling for the first factor in the Harman’s single‐factor test.

**Supporting Information 2** STROBE Statement‐Checklist.

## Data Availability

The data that support the findings of this study are available from the corresponding author upon reasonable request.
