# Supplementary material for: The Association of Fatalistic Determinism With Problematic Smartphone Use in Nurses: A Cross‐Sectional Study Assessing the Mediating Roles of Self‐Control and Grit
Source: J Nurs Manag. 2026 Aug 26;2026:6614190. doi: 10.1155/jonm/6614190 (PMC13507707; doi:10.1155/jonm/6614190)
Supplement: Supplementary file 1 [file JONM-2026-6614190-s001.docx]

**Supplementary materials for**

***‘The association of fatalistic determinism with problematic smartphone use in nurses: A cross-sectional study assessing the mediating roles of self-control and grit’***

**Table S1.** Total, direct, and indirect effects of the mediation models after controlling for sociodemographic factors

**Table S2.** Total, direct, and indirect effects of the mediation models after controlling for job stress

**Table S3.** Total, direct, and indirect effects of the mediation models after controlling for the first factor in the Harman’s single-factor test

**Figure S1.** Fatalistic determinism is linked to problematic smartphone use through the self-control → grit pathway (a) or the grit → self-control pathway (b) after controlling for sociodemographic factors

**Figure S2.** Fatalistic determinism is linked to problematic smartphone use through the self-control → grit pathway (a) or the grit → self-control pathway (b) after controlling for job stress

**Figure S3.** Fatalistic determinism is linked to problematic smartphone use through the self-control → grit pathway (a) or the grit → self-control pathway (b) after controlling for the first factor in the Harman’s single-factor test

**Table S1.** Total, direct, and indirect effects of the mediation models after controlling for sociodemographic factors

| Effect | Effect size (β) | Bootstrap SE | Bootstrap 95% CI |
| --- | --- | --- | --- |
| Model 1 (specifying self-control before grit) |  |  |  |
| Total effect (Fatalistic determinism → Problematic smartphone use) | 0.38 | 0.04 | [0.29, 0.47] |
| Direct effect (Fatalistic determinism → Problematic smartphone use) | 0.20 | 0.04 | [0.11, 0.28] |
| Indirect effect | 0.18 | 0.03 | [0.13, 0.25] |
| Fatalistic determinism → Self-control → Problematic smartphone use | 0.11 | 0.03 | [0.06, 0.19] |
| Fatalistic determinism → Grit → Problematic smartphone use | 0.03 | 0.01 | [0.01, 0.05] |
| Fatalistic determinism → Self-control → Grit → Problematic smartphone use | 0.04 | 0.01 | [0.01, 0.07] |
| Model 2 (specifying grit before self-control) |  |  |  |
| Total effect (Fatalistic determinism → Problematic smartphone use) | 0.38 | 0.04 | [0.29, 0.47] |
| Direct effect (Fatalistic determinism → Problematic smartphone use) | 0.20 | 0.04 | [0.11, 0.28] |
| Indirect effect | 0.18 | 0.03 | [0.13, 0.25] |
| Fatalistic determinism → Grit → Problematic smartphone use | 0.07 | 0.02 | [0.02, 0.12] |
| Fatalistic determinism → Self-control → Problematic smartphone use | 0.04 | 0.02 | [0.01, 0.08] |
| Fatalistic determinism → Grit → Self-control → Problematic smartphone use | 0.07 | 0.02 | [0.04, 0.11] |

*Note.* SE, standard error; CI, Confidence Interval.

**Table S2.** Total, direct, and indirect effects of the mediation models after controlling for job stress

| Effect | Effect size (β) | Bootstrap SE | Bootstrap 95% CI |
| --- | --- | --- | --- |
| Model 1 (specifying self-control before grit) |  |  |  |
| Total effect (Fatalistic determinism → Problematic smartphone use) | 0.35 | 0.04 | [0.27, 0.44] |
| Direct effect (Fatalistic determinism → Problematic smartphone use) | 0.20 | 0.04 | [0.11, 0.29] |
| Indirect effect | 0.15 | 0.03 | [0.10, 0.21] |
| Fatalistic determinism → Self-control → Problematic smartphone use | 0.09 | 0.03 | [0.04, 0.15] |
| Fatalistic determinism → Grit → Problematic smartphone use | 0.03 | 0.01 | [0.01, 0.05] |
| Fatalistic determinism → Self-control → Grit → Problematic smartphone use | 0.03 | 0.01 | [0.01, 0.06] |
| Model 2 (specifying grit before self-control) |  |  |  |
| Total effect (Fatalistic determinism → Problematic smartphone use) | 0.35 | 0.04 | [0.27, 0.44] |
| Direct effect (Fatalistic determinism → Problematic smartphone use) | 0.20 | 0.04 | [0.11, 0.29] |
| Indirect effect | 0.15 | 0.03 | [0.10, 0.21] |
| Fatalistic determinism → Grit → Problematic smartphone use | 0.06 | 0.02 | [0.02, 0.10] |
| Fatalistic determinism → Self-control → Problematic smartphone use | 0.03 | 0.02 | [0.01, 0.07] |
| Fatalistic determinism → Grit → Self-control → Problematic smartphone use | 0.06 | 0.02 | [0.03, 0.09] |

*Note.* SE, standard error; CI, Confidence Interval.

**Table S3.** Total, direct, and indirect effects of the mediation models after controlling for the first factor in the Harman’s single-factor test

| Effect | Effect size (β) | Bootstrap SE | Bootstrap 95% CI |
| --- | --- | --- | --- |
| Model 1 (specifying self-control before grit) |  |  |  |
| Total effect (Fatalistic determinism → Problematic smartphone use) | 0.28 | 0.04 | [0.19, 0.37] |
| Direct effect (Fatalistic determinism → Problematic smartphone use) | 0.20 | 0.04 | [0.11, 0.29] |
| Indirect effect | 0.08 | 0.02 | [0.04, 0.12] |
| Fatalistic determinism → Self-control → Problematic smartphone use | 0.03 | 0.02 | [0.01, 0.07] |
| Fatalistic determinism → Grit → Problematic smartphone use | 0.03 | 0.01 | [0.01, 0.05] |
| Fatalistic determinism → Self-control → Grit → Problematic smartphone use | 0.02 | 0.01 | [0.01, 0.03] |
| Model 2 (specifying grit before self-control) |  |  |  |
| Total effect (Fatalistic determinism → Problematic smartphone use) | 0.28 | 0.04 | [0.19, 0.37] |
| Direct effect (Fatalistic determinism → Problematic smartphone use) | 0.20 | 0.04 | [0.11, 0.29] |
| Indirect effect | 0.08 | 0.02 | [0.04, 0.12] |
| Fatalistic determinism → Grit → Problematic smartphone use | 0.04 | 0.02 | [0.01, 0.08] |
| Fatalistic determinism → Self-control → Problematic smartphone use | 0.02 | 0.01 | [0.01, 0.04] |
| Fatalistic determinism → Grit → Self-control → Problematic smartphone use | 0.02 | 0.02 | [0.01, 0.04] |

*Note.* SE, standard error; CI, Confidence Interval.

**Figure S1.** Fatalistic determinism is linked to problematic smartphone use through the self-control → grit pathway (a) or the grit → self-control pathway (b) after controlling for sociodemographic factors. Standardized regression coefficients were displayed in the path diagram; c, total effect; c’, direct effect. ^***^, *p* < 0.001; ^**^, *p* < 0.01; ^*^, *p* < 0.05.

**Figure S2.** Fatalistic determinism is linked to problematic smartphone use through the self-control → grit pathway (a) or the grit → self-control pathway (b) after controlling for job stress. Standardized regression coefficients were displayed in the path diagram; c, total effect; c’, direct effect. ^***^, *p* < 0.001; ^**^, *p* < 0.01; ^*^, *p* < 0.05.

**Figure S3.** Fatalistic determinism is linked to problematic smartphone use through the self-control → grit pathway (a) or the grit → self-control pathway (b) after controlling for the first factor in the Harman’s single-factor test. Standardized regression coefficients were displayed in the path diagram; c, total effect; c’, direct effect. ^***^, *p* < 0.001; ^**^, *p* < 0.01; ^*^, *p* < 0.05.
